# Characteristics and Functional Properties of Bioactive Oleogels: A Current Review

**DOI:** 10.3390/gels11010069

**Published:** 2025-01-16

**Authors:** Md. Jannatul Ferdaus, Niaz Mahmud, Sudipta Talukder, Roberta Claro da Silva

**Affiliations:** Food and Nutritional Sciences Program, Department of Family and Consumer Sciences, North Carolina Agricultural and Technical State University, Greensboro, NC 27411, USA; mferdaus@aggies.ncat.edu (M.J.F.); nmahmud@aggies.ncat.edu (N.M.); stalukder@aggies.ncat.edu (S.T.)

**Keywords:** oleogels, bioactive compounds, incorporation, delivery methods, bioactive oleogels

## Abstract

Oleogels have been a revolutionary innovation in food science in terms of their health benefits and unique structural properties. They provide a healthier alternative to traditional solid or animal fats. They have improved oxidative stability and nutritional value to maintain the desirable sensory qualities of lipid-based foods. Moreover, oleogels offer an ideal carrier for poorly water-soluble bioactive compounds. The three-dimensional structure of oleogels can protect and deliver bioactive compounds in functional food products. Bioactive compounds also affect the crystalline behavior of oleogelators, the physical properties of oleogels, and storage stability. Generally, different incorporation techniques are applied to entrap bioactive compounds in the oleogel matrix depending on their characteristics. These approaches enhance the bioavailability, controlled release, stability of bioactive compounds, and the shelf life of oleogels. The multifunctionality of oleogels extends their applications beyond fat replacements, e.g., food preservation, nutraceutical delivery, and even novel innovations like 3D food printing. Despite their potential, challenges such as large-scale production, cost efficiency, and consumer acceptance remain areas for further exploration. This review emphasizes the understanding of the relationship between the structure of oleogels and their functional properties to optimize their design in different food applications. It also highlights the latest advancements in bioactive oleogels, focusing on how they incorporate bioactive compounds such as polyphenols, essential oils, and flavonoids into oleogels. The impact of these compounds on the gelation process, storage stability, and overall functionality of oleogels is also critically examined.

## 1. Introduction

Oleogels are restructured semi-solid lipids prepared by transforming liquid oils into gels without chemical modifications or hydrogenation. The process requires dispersing a small amount of structuring agents (oleogelators) into liquid oils, which turns them into a gel-like phase [1]. This innovative technology allows the diverse use of unsaturated oils, such as plant-based oils, while maintaining solid fats’ physical and functional properties. Traditionally, solid fats like trans fats and saturated fats have been necessary in fat-based food processing to provide the desired structure, texture, and stability to the finished products [2].

These traditional solid or saturated fats (hydrogenated fats and palm oils) are associated with several negative health conditions, particularly cardiovascular diseases (CVDs), heart attack, obesity, type 2 diabetes mellitus (T2DM), and inflammation [3]. The World Health Organization (WHO) and the Food and Drug Administration (FDA) have implemented strict measures to reduce trans-fat consumption. The WHO recommends a national limit of trans-fat should be 2% of total fat in foods [4]. The FDA banned partially hydrogenated oils (PHOs) by declaring them no longer “Generally Recognized as Safe” (GRAS) [5]. The presence of trans-fat is needed to be listed on the Nutrition Facts labels of a food product. Further, the FDA issued a rule to remove outdated references to PHOs from regulations in 2023 [5]. Consequently, the FDA and WHO are encouraging the food industries to replace trans-fat with oils rich in polyunsaturated fatty acids (PUFAs) (e.g., safflower, corn, sunflower oils) or monounsaturated fatty acids (MUFAs) (e.g., canola, olive, peanut oils) [4,5].

Food industries have shown increased interest in incorporating bioactive compounds into products due to their potential to provide health benefits that extend beyond basic human nutrition [6]. Bioactive compounds, such as polyphenols; carotenoids; n-3, n-6, and n-9 fatty acids; and vitamins, have gained attention for their potential to reduce inflammation and oxidative stress, as well as to support cognitive health [7,8]. Consequently, they have become key ingredients in the development of functional foods [9]. However, as bioactive compounds can degrade during processing and storage, their incorporation into foods presents some challenges [10].

Oleogels offer a promising solution by enabling the replacement of unhealthy fats without compromising the texture, mouthfeel, or functionality of the final product [11,12,13,14]. In recent years, oleogels gained attention to developing food products that meet health guidelines and the growing consumer demand for low-fat options [12,14]. Additionally, oleogels have emerged as effective carriers for bioactive compounds, as they can entrap and stabilize these health-promoting substances within a structured lipid matrix [15]. Consequently, they prevent the degradation of bioactive compounds during food processing, storage, and particularly in digestion [16]. Incorporating bioactive oleogels into food products makes them a valuable substitute for saturated fats to promote overall well-being and prevent disease [10,17].

This review explores how oleogels act as carriers for bioactive compounds to enhance their stability in different food products and bioavailability in the digestive tract. It also examines the methods of incorporating bioactive compounds into oleogels and the nutritional benefits they offer. This review aims to identify challenges and opportunities for developing and commercializing bioactive-enriched oleogels in modern food products.

## 2. Preparation of Oleogels

Oleogelation is an alternative structuring technique to create a solid fat-like structure in edible oils by mimicking the fat crystal network. In this process, oleogelators are dissolved in the liquid oil at elevated temperatures with continuous stirring to ensure a uniform mixture. This is followed by rapid cooling to room temperature and subsequent storage at low temperatures (e.g., 4 °C) to facilitate the self-assembly of oleogelators into crystalline or tubular networks [18,19]. These added oleogelators form a three-dimensional structure that immobilizes the liquid oil and form oleogels [11,12,13]. The selection of oleogelators, such as waxes, monoglycerides, or ethyl cellulose, depends on their ability to form stable networks, maintain desirable textures, and meet food-grade standards [18,19,20]. Further, the oleogelators should solidify oils with minimal quantities, be cost-effective, require no additional processing, and possess GRAS status or approval for use in food [21,22]. However, oleogelator concentration, cooling rate, and oil type influence the rheological and textural properties of the resulting oleogels. Characterization techniques like microscopy, X-ray diffraction, rheological testing, and differential scanning calorimetry are employed to evaluate the structural and functional properties of oleogels [18]. This method provides a versatile platform for creating oleogels with stabilizing bioactive compounds, enhancing oxidative stability, and controlling lipid digestion in functional food systems [19,20].

### 2.1. Characteristics of Oleogelators

Oleogelators are the key structuring agents of liquid oil used to form oleogels. In bioactive oleogels, oleogelators not only provide the desired texture and stability but also take part in the carrying of bioactive compounds. Oleogelators can be broadly classified into two main categories based on their molecular weight: low-molecular-weight oleogelators (LMWOs) and high-molecular-weight oleogelators (HMWOs). LMWOs are small molecules that self-assemble into fibrillar, crystalline, or tubular structures to form a network that traps liquid oils. Common examples include waxes, fatty acids, alcohols, and phytosterols. HMWOs are large molecules, for example, polysaccharides and proteins, that form networks through chain entanglement or cross-linking [23,24].

#### 2.1.1. Low-Molecular-Weight Oleogelators

Natural waxes are widely used as oleogelators in food products. They crystallize to form a solid-like structure that can efficiently entrap oils and bioactive compounds [16]. According to Toro-Vazquez et al. [25], natural waxes may gel liquid oils at concentrations as low as 1–4 wt% by creating a three-dimensional network that traps the oil inside its pores and adsorbs it to the network’s surface. However, the chemical composition of natural waxes (e.g., the percentages of fatty alcohols, fatty acids, and hydrocarbon chains) depends on their sources, and the proportions of different chemical compounds influence the gelation behavior of waxes in the liquid oil phase. Moreover, the gelation behavior of natural waxes is also influenced by the composition of the liquid oils. Their saturation level is directly associated with the improvement of the oleogelation [26].

The benefit of using plant waxes in oleogels is that they are readily available, inexpensive, and of food-grade quality. Further, these wax-based oleogels can be used to formulate water-in-oil type structured emulsions that may or may not require emulsifiers [27,28]. However, among natural waxes, candelilla (CLW), carnauba (CRW), rice bran (RBW), sunflower (SFW), and beeswaxes (BW) have the highest interest for the applications in edible oleogels. Wax esters are the primary component in many waxes and are crucial for the high-temperature crystallization phase. CLW is known for its high wax ester content and low levels of hydrocarbons and fatty alcohols. This composition of CLW is responsible for its high thermal stability [29]. CRW contains a mix of wax esters, fatty alcohols, and hydrocarbons and produces robust crystalline structures [30]. According to Lim et al. [30], CLW oleogels maintained consistent viscoelastic properties up to ~40 °C, while CRW and BW oleogels showed more temperature sensitivity, with a decrease in elasticity and strength at higher temperatures. In addition, CLW oleogels had the lowest peroxide values, followed by CRW and BW oleogels [30]. In sunflower wax, esters (96.2% *w*/*w*) provide the structural backbone for oleogels [31]. On the other hand, hydrocarbons (e.g., n-alkanes) are significant in beeswax (27% *w*/*w*), which influence the crystallization and mechanical properties. For instance, SFW forms a continuous, porous network of large, needle-like crystals, whereas BW results in more compact, irregular structures with smaller pore sizes [31]. Moreover, a study by Wettlaufer et al. [31] showed that the characteristics of waxes as oleogelators could be changed with their hydrolysis. Hydrolysis breaks down wax esters into free fatty acids and free fatty alcohols. They participate in lower-temperature crystallization phases and often form mixed solid phases with higher-temperature crystallization. This mixed crystallization can enhance structural strength and influence the sensory properties of oleogels [31].

Long-chain fatty acids, fatty acid esters, glycerolipids, and sterol lipids can self-assemble into fibrous networks to form a semi-solid structure within liquid oils in oleogelation. Stearic acid (SA) is a saturated long-chain fatty acid that forms needle-like crystals that aggregate into networks. It has a high melting point (68–71 °C), which contributes to forming stable crystalline networks in oleogels [32,33]. The molecular self-assembly of SA facilitates the gelation process and is stabilized by hydrogen bonding and van der Waals interactions [33]. SA primarily exhibits stable “C polymorph” (the most stable crystalline form), which contributes to its ability to form strong crystalline networks in oleogels. Consequently, stearic acid enhances the thermal stability of oleogels. Moreover, the firmness and oil binding capacity of oleogels increases with the proportion of stearic acid in the gelator mixture [32,33].

Glycerol monostearate (GMS) is also widely used as a structuring agent in oleogelation. It is soluble in hot organic solvents but insoluble in aliphatic solvents. GMS has a high melting point (73–81 °C), hydrogen bonding capability, and self-assembly properties [34,35]. GMS-based oleogels consistently show needle-like crystals regardless of the type of oil used. However, the kind of oil significantly influences the behavior of GMS. It forms strong gels with high crystallinity in long-chain unsaturated oils (e.g., sunflower oil). On the other hand, it struggles to form a stable network in medium-chain saturated oils (e.g., coconut oil) [34]. The addition of amphiphiles in GMS-oleogels increases gelation time and crystal aggregation. Consequently, it produces softer textures but more stable gels. For instance, the addition of sorbitan monooleate (span 80) and pluronic F68 as an emulsifier in GMS-oleogels increases gel strength and stability without significantly affecting gel stiffness, respectively [35].

Lecithin, a soybean-derived glycerophospholipid, is also used as an oleogelator. Its amphiphilic nature makes it an effective emulsifier and stabilizer in oil-based systems [21,36]. The self-assembled structures of lecithin in oils play a key role in stabilizing the oleogel network. Lecithin enhances the structural stability of oleogels by promoting the formation of a three-dimensional network through interactions with other oleogelators. It improves the spreadability and smoothness of oleogels, which is suitable for their applications in spreads and margarine substitutes. Lecithin is effective in entrapping and delivering bioactive compounds within oleogels due to its amphiphilic properties [21,36,37].

Plant-derived phytosterols are well known for lowering cholesterol levels. These compounds are sterol molecules containing a sterol ring, with a hydroxyl group at C-3 and a hydrophobic aliphatic side chain [38,39]. Phytosterols have high melting points and form crystalline structures in the oleogelation process through hydrogen bonding. X-ray diffraction studies indicate the presence of β and β′ polymorphic crystals in phytosterol-oleogels and reversible transitions upon temperature changes. Below 65 °C, they exhibit characteristics like a solid and transform back into a gel upon cooling down. Studies indicate that incorporating phytosterols in oleogels can assist in reducing LDL cholesterol levels with the consumption of plant sterols (1.5–3.0 g per day), leading to a decrease in LDL levels by 5–19% [40]. These studies suggest that oleogels containing phytosterols could help reduce fat intake and offer health benefits through cholesterol reduction.

#### 2.1.2. High-Molecular-Weight Oleogelators

Ethyl cellulose (EC) is a versatile oleogelator capable of structuring a wide range of oils into stable semi-solid systems with thermal stability and oxidative protection. EC is a chemically modified linear polysaccharide derived from cellulose with ethoxyl groups replacing hydroxyl groups (48–49.5% ethoxyl content) [41,42,43]. The degree of substitution (approximately 2.5 ethoxy groups per glucose unit) imparts solubility in organic solvents and lowers its glass transition temperature (Tg), ranging from 125 °C to 130 °C [41,44]. That is why the dissolution of EC during oleogel preparation requires a temperature above its Tg, which is up to 150 °C [44]. At that melting temperature, EC forms physical gels via non-covalent interactions (hydrogen bonding), which are thermo-reversible and highly dependent on temperature [43]. EC oleogels form via polymer entanglement after dissolving in hot oil above Tg, and their re-cooling forms a three-dimensional gel network. In the process of oleogelation using EC, a strong crystalline network is created that traps oil effectively and prevents oil leaks during the storage period [42,43]. Nevertheless, the strength of the gel is subject to factors such as the polarity of the oil, which significantly affects how well EC can form the gel network. Oils that have polarity or contain additives play a key role in enhancing the gel strength by promoting interactions between the polymer and solvent [42]. Therefore, adjusting the gelation temperatures, EC concentration, and surfactants can help tailor EC oleogels to meet the needs of various food products.

Hydroxypropyl methylcellulose (HPMC) is another modified cellulose derivative that has recently gained significant attention as an oleogelator due to its unique amphiphilic nature. It has substituted hydroxyl groups with hydroxypropyl and methoxy groups [45]. Hence, it shows both hydrophilic and hydrophobic properties. HPMC forms a three-dimensional gel network by binding oil droplets within its matrix. The HPMC-oleogel is produced by following the emulsion-templated method. In this method, water is removed from an oil-in-water emulsion to produce a solid oleogel [46]. However, HPMC-based oleogels have exceptional thermal stability, and they are characterized by their temperature-independent solid-like behavior. This means that even under a wide range of temperatures, they retain their gel network, unlike many traditional fats, and resist thermal breakdown [45]. The strong three-dimensional network formed by HPMC molecules immobilizes the oil phase effectively and is responsible for their thermal stability. HPMC-oleogels demonstrate higher firmness and even maintain structural strength under varying shear stress (shear resistance) compared to animal fats. The structural stability of HPMC-oleogels offers high oil-binding capacity and less oxidative degradation, which further results in stable and long-lasting gels [46,47]. These characteristics make them suitable for various food applications and extend the product’s shelf life.

Proteins are food-grade ingredients and promising oleogelators. They offer unique structural and functional benefits in the development of oleogels. Unlike traditional oleogelators (e.g., waxes or polymers), their amphiphilic nature allows interactions with both hydrophilic and hydrophobic components. Animal-based proteins show higher solubility and more homogeneous networks compared to plant-based proteins [48]. Proteins can form various self-assembled structures (e.g., aggregates, fibrils, or networks) that are adaptable to different oil systems, and protein-based oleogels have significant potential as solid fat replacers in food systems. Whey protein isolates (WPI) are globular proteins that undergo denaturation and aggregation when heated. At pH 5.7 and 85 °C, WPI exposes their hydrophobic side chains that contribute to aggregation and gel network formation and are stabilized by covalent disulfide bonds and non-covalent hydrophobic interactions [49]. The heat-set protein aggregates in water are transferred from water to oil using the solvent exchange method to ensure the preservation of their structure [48]. The proteins form three-dimensional networks through aggregation or particle interactions, and those networks create oleogels by immobilizing liquid oil [50]. The gel strength is strongly correlated with protein aggregate size and hydrophobicity. However, smaller and more hydrophilic aggregates form the strongest gels because their small size enables a uniform and tight network, while their hydrophilic nature promotes interactions among the aggregates. Conversely, larger and more hydrophobic aggregates struggle to form strong gels because their size limits the network density, and their hydrophobic nature reduces the aggregate-to-aggregate bonding needed for a cohesive gel [48]. That is why oleogels with smaller protein aggregates show higher oil binding capacity than the oleogels with larger aggregates. Further, heat treatment enhances protein–protein interactions and helps to produce firmer and more cohesive oleogels. However, protein oleogels can maintain their structure across temperature cycles due to their thermo-reversible behavior [49]. Protein’s ability to form strong gel networks in oil demonstrates their potential as sustainable alternatives to traditional oleogelators.

## 3. Incorporation of Bioactive Compounds into Oleogels

**Figure 1:**
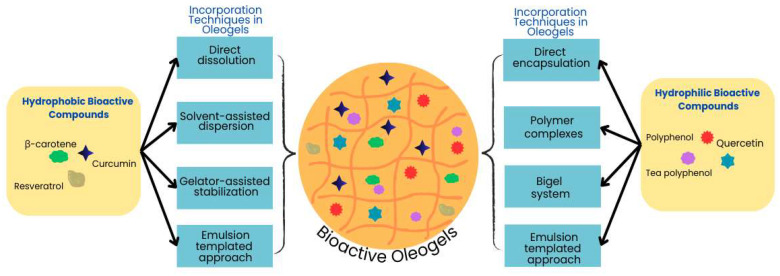
Incorporation techniques of hydrophobic and hydrophilic bioactive compounds in oleogels.

The incorporation of bioactive compounds into oleogels represents an innovative approach to the development of functional and nutritionally enriched lipid-based systems. Bioactive compounds, e.g., polyphenols, carotenoids, vitamins, and omega fatty acids, are well known for their health-promoting properties, for example, antioxidant, anti-inflammatory, and antimicrobial activities. Bioactive oleogels are an effective carrier for these compounds due to their fat solubility. The three-dimensional structure of oleogels traps and protects these compounds from damage and oxidation while controlling how they are released. Further, bioactive compounds can regulate the crystalline structure and network density of the oleogelators, hence changing the physical properties and stability of the final oleogels [37,51,52]. For instance, curcumin incorporation produces stronger and more cohesive gel networks in wax-based oleogels by enhancing their crystalline morphology [53,54]. Their addition enhances the nutritional value of oleogels and introduces novel functional properties. However, there are different approaches to the addition of bioactive compounds into oleogels, considering their hydrophobicity or hydrophilicity (Figure 1).

### 3.1. Hydrophobic Bioactive Compounds

Hydrophobic bioactive compounds are incorporated in oleogels followed by (a) direct dissolution, (b) solvent-assisted dispersion, (c) gelator-assisted stabilization, or (d) an emulsion-templated approach [53,55,56,57].

#### 3.1.1. Direct Dissolution

The direct dissolution method is a straightforward and widely used approach for incorporating hydrophobic bioactive compounds into oleogels. Bioactive compounds are dissolved in the liquid oil phase prior to the gelation process [55]. The liquid oil is preheated (if necessary) carefully to create a homogeneous medium and avoid thermal degradation of sensitive bioactive compounds. Then, the bioactive-enriched oil is mixed with the chosen oleogelators. At cooling, the mixture undergoes the gelation process, and depending on the oleogelator used, this process involves solidification or polymer entanglement. For instance, Dent et al. [53] prepared curcumin (hydrophobic compound)-loaded oleogels with corn oil and rice bran wax following the direct dissolution method. Curcumin was added to the pre-weighted corn oil and heated to 150 °C. The whole mixture was then agitated at 350 rpm for 10 min to dissolve the curcumin completely. The RBW was added to the curcumin-enriched oil. Then, the wax formed a crystalline network that traps the oil and curcumin upon cooling [53].

#### 3.1.2. Solvent-Assisted Dispersion

Bioactive compounds that are poorly soluble in oil are dissolved in a compatible organic solvent (e.g., ethanol or acetone) and then mixed with liquid oil in a solvent-assisted dispersion method of bioactive oleogel production. The uniform mixing of the oil phase and the bioactive solvent is done by stirring or sonication. The solvent is evaporated before or during the gelation process to ensure uniform distribution of bioactive compounds into the oleogel matrix. However, structuring agents such as waxes or GMS are added to the bioactive-enriched oil, and the mixture is heated to dissolve the structuring agent. At cooling, the oleogelators form a three-dimensional network that entraps the liquid oil and bioactive compounds [56].

#### 3.1.3. Gelator-Assisted Stabilization

Surfactants and co-oleogelators are widely known as essential components in oleogel production. Co-gelators are additional substances that work alongside the primary oleogelator to enhance or modify the oleogelation process [58,59]. They enhance the stabilization of hydrophobic bioactive compounds in the oleogel matrix. They are applied together to encapsulate bioactive substances. Surfactants promote uniform dispersion, while co-gelators produce a stable matrix to encapsulate the bioactive components within the oleogels [58,59]. However, the combination of these two compounds diminishes the phase separation and oxidation of oleogels. Their chemical compositions and structural features of surfactants and co-gelators provide efficient solubilization, encapsulation, and protection of bioactive substances. Surfactants contain hydrophilic head groups and hydrophobic tail groups, which reduce the interfacial tension between hydrophobic bioactive chemicals and the oil phase, hence facilitating their uniform dispersion [58,59]. For instance, polyglycerol polyricinoleate (PGPR) (surfactant) improves the dispersion of resveratrol in oleogels based on monoglycerides [57]. PGPR consists of polyglycerol esters of polyricinoleic acid, which offer superior emulsifying capabilities and elevated oil solubility [60]. Further, sorbitan monostearate has hydrophobic long-chain fatty acids linked to a sorbitan backbone. The characteristics of sorbitan monostearate enhance oil-phase compatibility and stabilize bioactive substances at the molecular level. Furthermore, co-gelators modify the polarity of the oil phase to make it more compatible with hydrophobic bioactive compounds. For example, mono-diglycerides (co-gelators) influence nucleation and create a denser network that immobilizes the oil phase and encapsulates bioactive compounds [59].

#### 3.1.4. Emulsion-Templated Approach

The emulsion-templated approach is another effective approach to incorporate bioactive compounds (hydrophobic or hydrophilic) in oleogels. It utilizes oil-in-water (O/W) or water-in-oil (W/O) emulsions as foundation. After that, the emulsions are transformed into oleogels by removing the continuous phase (usually water). Generally, a high-internal-phase emulsion is created and stabilized with biopolymers [55]. However, bioactive compounds are incorporated into either the dispersed or continuous phase. Adding stabilizers and emulsifiers in emulsion ensures the uniform distribution of bioactive compounds. The emulsion is dried or freeze-dried to remove the continuous phase (water), leaving behind a 3D gel network that encapsulates the bioactive compounds [61]. The drying process is necessary to ensure that the bioactive compounds remain intact and uniformly distributed. The resulting oleogel retains the oil phase. Consequently, it ensures minimal oil leakage and provides a stable matrix for bioactive compounds. Santos et al. [55] prepared high-internal-phase emulsions stabilized by sodium caseinate–quercetin (SC-Q) complexes. The aqueous phase (water and SC-Q) was removed from sunflower oil by oven drying at temperatures between 80 and 120 °C. This method enhanced the stability of quercetin while maintaining oil-binding capacity and reducing lipid oxidation [55]. In another study, β-carotene was dissolved in corn oil and incorporated into an oleogel matrix with monostearin and Span 20 (sorbitan monolaurate). The oleogel was emulsified with Tween (polysorbate) 20, 40, 60, and 80 (emulsifiers) and processed using high-pressure homogenization to form a nanoemulsion [61]. This approach significantly improved the bioaccessibility and stability of β-carotene and ensured better in vivo performance. The resulting oleogels using this method exhibit desirable textural and thermal properties and are adaptable for both hydrophilic and hydrophobic bioactive compounds [55,61,62].

### 3.2. Hydrophilic Bioactive Compounds

The incorporation of hydrophilic bioactive compounds into oleogels poses unique challenges due to their poor solubility in oil phases. In recent years, different techniques have been employed to successfully incorporate these compounds while maintaining their stability and functional properties. These methods include (a) direct encapsulation, (b) polymer complexes, and (c) the bigel system [37,63,64,65,66].

#### 3.2.1. Direct Encapsulation

Hydrophilic bioactive compounds are inserted directly into the oleogel using carrier systems or gelators. They are encapsulated into solid gelator matrices, such as beeswax, or co-encapsulated with hydrophobic compounds [37,63]. The encapsulation ensures uniform dispersion within the oleogel. It protects bioactive compounds from oxidation, thermal degradation, and light exposure. Shang et al. [37] used beeswax as a gelator to directly introduce hydrophilic bioactive compounds like tea polyphenols into the oleogel matrix. Tea polyphenols were dissolved in an aqueous phase and mixed with beeswax and soybean lecithin at 80 °C [37]. A water-in-oil emulsion was formed using high-shear mixing and was freeze-dried at −20 °C for 48 h to remove water to create a polyphenol-loaded gelator. The polyphenol-loaded gelator was mixed with soybean oil at 80 °C and cooled to solidify into oleogels [37]. In another study, Meng et al. [63] applied ferritin (a cage-like protein) to directly encapsulate hydrophilic epigallocatechin gallate. The bioactive compound was encapsulated in apo-phytoferritin, which was derived from red bean seeds. Encapsulation was achieved using pH transitions (2.0 to 6.7) and urea-induced channel expansion to incorporate the bioactive compounds into ferritin’s hollow structure [63]. However, direct encapsulation techniques offer versatile and effective methods to incorporate bioactive compounds into oleogels. These approaches enhance the stability and bioavailability of bioactive compounds and make them suitable for functional food. The selection of gelators and encapsulation conditions plays a pivotal role in achieving desirable characteristics of the final oleogels [15,63].

#### 3.2.2. Polymer Complexes

The interaction between different polymers produces complexes that can enhance the structural consistency of oleogels. Proteins such as egg white protein or gelatin interact with polysaccharides like xanthan gum or flaxseed gum through hydrogen bonding, electrostatic interactions, or covalent linkages to produce complexes [64,67]. These complexes can encapsulate hydrophilic bioactive compounds and provide them with stability and protection against degradation. For instance, the egg white protein, gallic acid, and xanthan gum complex were utilized by Yan et al. [67] to incorporate gallic acid in the oleogel system. The mixture was dialyzed to remove unreacted components and then freeze-dried to produce a powdered complex. The complex was dissolved in water and emulsified with corn oil at a ratio of 4:1 (*v*/*v*). The emulsion was then again freeze-dried and sheared to form the oleogel. A higher concentration of the complex (0.2–1.0%, *w*/*w*) improves the emulsification activity, gel strength, and oxidative stability of the oleogels by reducing droplet size and increasing negative zeta potential [67]. Another study by Pan et al. [64] used gelatin and xanthan gum or pectin to combine proanthocyanidins in oleogel. Gelatin and proanthocyanidins were dissolved in the water and mixed to form colloidal complexes. Polysaccharides such as xanthan gum or pectin were added to stabilize the emulsion. The water phase was removed by freeze-drying to create a dry matrix that was later mixed with soybean oil to form the oleogel. However, the inclusion of proanthocyanidin improves oxidative stability and enhances oleogel strength [64].

#### 3.2.3. Bigel System

Hydrophilic bioactive compounds can be incorporated into oleogels by utilizing the bigel system, which is a combination of hydrogels and oleogels. It could also be used as a delivery medium for hydrophobic bioactive compounds. Bigels can exist in three configurations: hydrogel-in-oleogel, oleogel-in-hydrogel, or bi-continuous systems, which depend on the distribution of phases [65,66,68]. Hydrogels are prepared using natural or synthetic polymers, e.g., kappa-carrageenan, whey protein concentrate, or potato protein isolate [65,66]. These gelling agents provide the matrix for incorporated hydrophilic compounds. After that, bioactive compounds such as polyphenols or vitamins are dissolved in the aqueous phase before gelation. The hydrogel phase is combined with the molten oleogel (canola-oil-based monoacylglycerol-oleogel) phase under controlled conditions [66]. Further, bigels prepared with potato-protein-based hydrogels and candelilla wax oleogels demonstrated improved bioaccessibility and stability of curcumin [65]. High shear mixing or sonication ensures uniform distribution of the hydrogel within the oleogel matrix. However, the temperature, shear rate, and oleogel/hydrogel ratio are critical parameters to ensure the functionality of the bigels [66,68,69]. Moreover, encapsulation efficiency can be further improved by reducing hydrogel particle size by applying ultrasonication or high-shear mixing [66].

Bigels and oleogels offer distinct advantages and limitations that make them suitable for different applications. Due to their dual-phase structure, bigels excel in encapsulating hydrophilic and hydrophobic bioactive compounds [70,71]. They also allow for the creation of unique textures tailored to specific products, such as low-fat spreads and desserts. On the other hand, oleogels are simpler to prepare and are highly effective in stabilizing lipid-soluble bioactive compounds, making them more suitable for lipid-rich food systems and applications requiring oxidative stability [14,53,55]. However, bigels may face phase interaction and processing complexity challenges, while oleogels are limited to lipid-based compounds [12,13,71]. The choice between bigels and oleogels depends on the intended application, with bigels being ideal for foods requiring hydrophilic compound delivery or novel textures and oleogels excelling in lipid stabilization.

## 4. Recent Advances in Bioactive Oleogels

The development of bioactive oleogels has been a great innovation in functional food systems, which enhance both the nutritional and structural properties of food products. The three-dimensional structure of oleogelators can entrap bioactive compounds. Consequently, they improve the solubility and stability of bioactive compounds. Further, bioactive oleogels enhance the bioavailability of incorporated bioactive compounds by promoting their absorption in the digestive tract. Furthermore, oleogel enables the controlled release of bioactive compounds to ensure their consistent therapeutic effects. Recent research highlights the different formulations of bioactive oleogels that combine different oleogelators, liquid oils, and bioactive compounds to optimize functionality and health benefits. Table 1 summarizes recent advances in bioactive oleogels and their application in different food products.

Tea polyphenols (TP) are a group of bioactive compounds derived from tea leaves. They are well-known for their antioxidant properties and different health benefits. However, the hydrophilic nature of TP limits their incorporation into lipid-based systems. TP-loaded oleogels have been developed to stabilize and enhance their functional properties. Generally, direct encapsulation and dissolution or emulsion-based templated systems are applied to encapsulate TP within the oleogel matrix (Table 1) [37,52]. This process involved dissolving TP in the aqueous phase at a concentration of 30 mg/mL (equivalent to 0.053% in the final oleogel) and combining it with gelators such as beeswax or glyceryl monostearate (GMS). Freeze-drying stabilizes the TP within the gelator matrix and ensures uniform dispersion and protection. In addition, curcumin (0.1%) was added to the soybean oil (liquid phase) [52]. Then, the TP-loaded gelators were mixed with soybean oil to form a stable oleogel. However, the incorporation of TP and curcumin significantly enhanced the oxidative stability of the oleogels by effectively reducing peroxide values (PV) and *p*-anisidine values (*p*-AV) during storage. Moreover, TP addition to the oleogel slightly weakened the crystalline network. These characteristics could be described by the interaction between TP and oleogelators. TP interacts with the hydrophilic groups of gelators like soybean lecithin or GMS through hydrogen bonding. These interactions reduce the gelators’ crystallization efficiency by altering their self-assembly behavior. Consequently, the presence of TP causes a reduction in the size and aggregation of gelator crystals [37,52]. Further, polarized light microscopy (PLM) images show that TP-loaded oleogels have fewer and less densely packed crystals compared to control oleogels. However, the melting profiles and gelation behaviors were not significantly affected, which proved that oleogels maintained their essential rheological and thermal characteristics [37,52].

β-Carotene (carotenoid) is a fat-soluble antioxidant that also helps in boosting immunity and reducing the risk of chronic diseases. However, its vulnerability to light exposure, heat, and oxygen limits its direct use in food products. Oleogels offer a protective way of stabilizing and delivering β-carotene in food applications (Table 1). Several techniques have been adopted to incorporate β-carotene in oleogels [51,56,76]. In different studies, β-carotene was added to the oleogels at levels ranging from 0.025% to 0.4% [51], 0.02% [56], or 1% (*w*/*w*) [76] by dissolving in the liquid oil or applying gelator-assisted stabilization [76]. The results show that β-carotene has a strong impact on the properties of the oleogels. β-Carotene significantly enhances oxidative stability by reducing peroxide values and protecting the oil phase from degradation [51,56]. Further, FTIR analysis proved that β-carotene improves the thermal stability of oleogels by forming complexes with the oleogelator molecules [51]. However, β-carotene disrupts the oleogel network at higher concentrations, which causes a softer texture and lower mechanical strength of oleogels [51]. Rheological analysis also proved the reduced viscosity and elasticity of oleogels with higher β-carotene concentration [56]. However, the disruption of the crystalline network in oleogels at higher β-carotene concentrations can be attributed to its interaction with the gelator components. β-Carotene (a pigment) does not directly participate in forming the three-dimensional crystalline network of oleogels. It alters the alignment and packing of gelator molecules, hence reducing the crystal density and size [51]. This interaction results in a softer texture and lower mechanical strength of the oleogels. Despite these effects, β-carotene enhanced the overall uniformity and improved bioavailability [56]. A zein-based oleogel system showed that encapsulation extended the retention time of β-carotene in the gastrointestinal tract and increased absorption efficiency during digestion [76].

Curcumin is the bioactive compound of turmeric and is widely recognized for its potent antioxidant, anti-inflammatory, and anticancer properties. However, its low water solubility, limited bioavailability, and rapid breakdown during digestion limit its application in functional food systems. Curcumin-loaded oleogels can enhance their stability in functional foods and bioavailability in the digestive tract. The preparation of curcumin-loaded oleogels follows different techniques based on the oleogelators and liquid oil phase (Table 1). The incorporation techniques of curcumin and its impact on the characteristics of oleogels have been reported in some previous studies [53,78,79,83]. Those studies used rice bran wax and beeswax as the oleogelator in different plant-based oils. Curcumin was incorporated at 0.1% (*w*/*w*) [53], 0.4% (*w*/*w*) [73], and 0.2% (*w*/*w*) [78,83] by dissolving it into the oil phase (direct dissolution) or 0.1%, 0.5%, and 1% (*w*/*w*) by applying an emulsion-templated approach [79,84]. The results show that curcumin enhances the oxidative stability of the oleogels and protects the liquid oils from oxidative degradation during storage [73,78,79]. However, curcumin slightly disrupts the oleogel network and leads to a soft texture and reduced mechanical strength at higher concentrations (e.g., 0.5%) [79]. This happened due to curcumin’s interaction with the self-assembly of oleogelator molecules, which altered their crystalline structure and rheological properties [53]. Curcumin-loaded oleogels demonstrate improved delivery and intestinal absorption. For example, the rice bran wax oleogels showed a significant increase in curcumin bioaccessibility [53]. Similarly, beeswax oleogel exhibited high curcumin bioaccessibility (>55%) regardless of the plant-based oil type, which is attributed to the micellar solubilization of curcumin during digestion [78]. These findings emphasize the potential of oleogels to improve the absorption of lipophilic bioactive compounds like curcumin.

Quercetin is a natural flavonoid in fruits, vegetables, and plants. It is known for its antioxidant, anti-inflammatory, and anticancer properties. Nevertheless, its limited water solubility and susceptibility to physiological degradation limit its incorporation in functional foods. In one study, quercetin was incorporated into high-internal-phase emulsions (HIPEs) stabilized by sodium caseinate–quercetin (SCQ) complexes [55]. These emulsions had 80% sunflower oil and 20% aqueous phase (containing SCQ), which were dried to form oleogels. The quercetin concentration ranged from 0.25 to 2.5 mmol/L in SCQ complexes. The preparation process emphasized the creation of covalent bonds between sodium caseinate and quercetin to enhance interfacial properties and oxidative stability during drying [55]. Zhang et al. [74] reported the direct dissolution of quercetin (0.1%, *w*/*w*) in high oleic sunflower oil with candelilla wax at 3%. Another study utilized a bigel system combining a candelilla-wax-based oleogel and a fish gelatin hydrogel. The oleogel was prepared using 3% (*w*/*w*) candelilla wax and quercetin (0.1%, *w*/*w*) in high oleic sunflower oil [70]. The presence of quercetin significantly impacted the physicochemical properties of the oleogels. Quercetin enhanced oxidative stability by reducing the formation of hydroperoxides and secondary oxidation products (malondialdehyde) in HIPE-derived oleogels [55]. Similarly, the bigel system showed quercetin retention and controlled release under gastrointestinal conditions. Quercetin also improved the bioavailability of oleogels. Moreover, higher ratios of hydrogel promote quercetin release. Xie et al. [70] reported that quercetin release increased from 2.40% to 11.08% as the oleogel fraction decreased from 70% to 30%. However, quercetin-loaded oleogels are a promising strategy for stabilizing and delivery of quercetin. These systems not only protect quercetin from degradation but also improve its oxidative stability, bioavailability, and controlled release.

Resveratrol is a natural polyphenol-rich in grapes and berries. Oleogels provide an efficient medium for delivering resveratrol (Table 1). Its application in food formulations has been hampered by its poor solubility in both water and oils [77,80,81]. Oleogels have emerged as potent carriers for resveratrol due to their structured lipid matrix. However, different approaches have been utilized to prepare resveratrol-loaded oleogels [57,77,80,81]. Qiu et al. [57] incorporated resveratrol in oleogel by applying gelator-assisted stabilization. Oleogelators, like rice bran wax, ethyl cellulose, and a mix of β sitosterol and lecithin, are heated to their melting points along with oils such as soybean and peanut oil in order to create a structure for trapping resveratrol at 0·5%. This method of using co-gelators ensures the formation of a matrix within the oleogels [57]. The emulsion-templated approach has been explored by Kavimughil et al. [81] and Wang et al. [80]. The authors initially added resveratrol to the oleogel by dissolving it in oil while creating an oil-in-water emulsion. The emulsion was stabilized using a mix of rice bran wax or gelatin and different amounts of gellan gum. After emulsifying it, the mixture was freeze-dried to eliminate the water phase and form a resveratrol-loaded oleogel [80,81]. Furthermore, resveratrol could also be included in the oleogel through the direct dissolution approach [77]. Adding resveratrol to these systems is usually done at concentrations to ensure it is effectively included and stabilized in the mixtures. Research has shown that even small amounts of resveratrol (ranging from 0.2–0.5%of the oil weight) can significantly improve the oil binding capacity, thermal properties, and viscoelasticity of oleogels [57,77,80]. The inclusion of resveratrol has an impact on both the functional aspects of oleogels by enhancing their hardness, oil retention capabilities, and oxidative stability by altering the crystalline structure of oleogelators. Furthermore, incorporating resveratrol into oleogels has been demonstrated to improve its absorption during simulated digestion studies. These characteristics are also affected by the type of oil and oleogelator utilized. For instance, oleogels derived from soybean oil generally display resistance to oxidation when compared to those made from peanut oil [57,77].

Polyphenols are a diverse class of bioactive compounds found in plants. They are recognized for their remarkable health benefits. Recent advancements in oleogel technology produced a potential way of encapsulating polyphenols within a structured lipid matrix (Table 1). Polyphenol-loaded oleogels were produced by incorporating polyphenols into polymer complexes. The polymer complexes were prepared with egg white protein and xanthan gum or gelatin and flaxseed gum [67,82]. The concentration of polyphenols in these systems ranged from 0.2% to 1.0% (*w*/*v*) [67] or 0.075% to 0.3% (*w*/*v*) [82]. Further, a study by Martinović et al. [75] added polyphenols to the oleogel system in the form of a mustard seed extract. The extract was dissolved in ethanol, followed by solvent-assisted dispersion. Then, the mixture was added to the sunflower oil to prepare the oleogel [75]. The results showed that polyphenols impact the structural and functional properties of oleogels by influencing the crystalline network and viscoelastic behavior. Studies report improved hardness, oil-binding capacity, and antioxidant activity in polyphenol-enriched oleogels compared to their plain counterparts [67,82]. Additionally, these oleogels exhibit superior oxidative stability due to the synergistic interaction between polyphenols and oleogelators. For example, the inclusion of gallic acid in egg white protein and xanthan-gum-based oleogels resulted in stronger gel networks and reduced peroxide value [67]. However, the type of liquid oil also plays a critical role in the formation and functionality of polyphenol-loaded oleogels. For example, soybean oil and corn oil have been widely used due to their high unsaturated fatty acid content. Incorporation of polyphenols decreases their oxidative degradation and extends the shelf life of oleogels [75,82].

The unique structural and functional properties of bioactive oleogels enable their application across a wide range of food products, e.g., baked goods, dairy alternatives, meat products, and confectionery (Table 1) [55,56,72,83,84,85]. Pound cakes made with quercetin [55], turmeric extracts (curcumin) [75], or vitamin E [85] enriched oleogels showed significant potential to enhance the nutritional and functional properties of pound cakes. The replacement of butter with turmeric-extract- or vitamin E-incorporated oleogels resulted in a noticeable reduction in hardness. The addition of bioactive oleogels increased the porosity of pound cakes by promoting air entrapment during mixing and baking. This softer texture and porosity are attributed to the structured lipid matrix and the functionality of the bioactive components [84,85]. Further, quercetin-based oleogels achieved a high specific volume in pound cakes, which suggests an effective aeration and structural integrity of oleogels [55]. Vitamin E-incorporated oleogels demonstrated better moisture retention in pound cakes compared to conventional fat sources. This is essential for extending shelf life and maintaining freshness during storage [85]. In addition, the application of bioactive oleogel in muffins also showed enhanced physicochemical characteristics [56]. Moreover, the application of bioactive oleogels in meat products also showed significant development in terms of their physical and functional properties [72,83]. The inclusion of curcumin in oleogels reduced their hardness compared to those made with traditional animal fat [83]. Oyom et al. [72] reported that oleogel coatings with thyme essential oil maintained the crust’s crispness and chewiness in fried chicken nuggets. This was due to the structural flexibility imparted by the oleogel matrix. Curcumin and thyme essential oil significantly reduced lipid oxidation by decreasing thiobarbituric acid reactive substances (TBARS) levels and peroxide values (PV) in pork burgers and chicken nuggets during storage and cooking [72,83]. However, the incorporation of bioactive compounds into oleogels offers a potential approach to enhance the nutritional, functional, and sensory properties of food products. Bioactive compounds like curcumin, vitamin E, quercetin, thyme essential oil, etc., enrich products with antioxidants and promote healthier lipid profiles by increasing polyunsaturated fatty acids (PUFAs).

## 5. Effect of Bioactive Compounds on the Physical Properties of Oleogels

**Figure 2:**
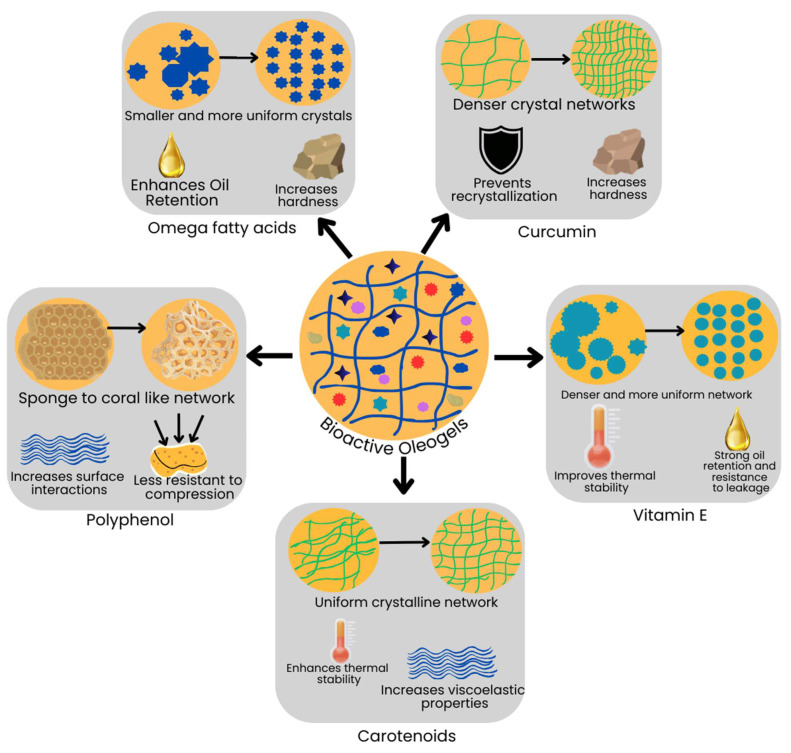
Effect of bioactive compounds on the physical properties of oleogels.

The incorporation of bioactive compounds like polyphenols, carotenoids, omega fatty acids (n-3, n-6, and n-9), and vitamins within oleogels creates unique interactions that influence the gel’s structural, mechanical, and nutritional properties (Figure 2). Polyphenols are known for their strong antioxidant activity. Polyphenols (e.g., catechins, quercetin, curcumin) interact with oleogelators to enhance the oxidative stability of the gel. Oleogelators like wax-based or phytosterol-based systems can encapsulate polyphenols, protecting them from oxidation. However, sometimes, these stronger bonds of oleogels may reduce the bioavailability of polyphenols during digestion. A previous study by Giacintucci et al. [86] focused on oleogel preparation with extra virgin olive oil and ethylcellulose and their interaction with polyphenols. In this study, atomic force microscopy revealed that as polyphenol content was reduced from olive oil, the oleogel structure transitioned from a sponge-like to a coral-like pattern. This means the oleogel network became more irregular, with uneven and extended pores. This demonstrates the role of oil polarity and polyphenols in maintaining oleogel network stability [86]. Another study by Ciuffarin et al. [87], based on calorimetric analysis, also reported that polyphenols play a key role in stabilizing the oleogel network. The researchers employed differential scanning calorimetry (DSC) to measure the thermal transitions and melting enthalpy of oleogels prepared with varying polyphenol levels. DSC revealed broader melting peaks at lower temperatures for oleogels without polyphenols, indicating weaker networks. Additionally, the presence of polyphenols increased the melting temperature and stabilized the network by creating additional junction points in the crystalline structure. For instance, in monoglyceride-based oleogels, reduced polyphenol content resulted in a more irregular and weaker network prone to melting, while in rice bran wax-based oleogels, polyphenols contributed to the formation of two distinct crystal fractions, providing enhanced structural stability. Further, PLM showed that polyphenols influence crystal morphology, transitioning from irregular structures to more organized networks. These findings underline the importance of combining calorimetric and microscopic techniques to explain the structuring role of polyphenols in oleogels [87].

Fat-soluble carotenoids are captured effectively into a lipid-based oleogel matrix. Oleogels with waxes or sterol esters provide structural stability to these carotenoids, preserve their bioactivity, and protect them from heat degradation [51]. This feature is very crucial for color-rich foods that need to retain bioactive compounds. However, increasing β-carotene content in oleogels reduced both their hardness and springiness (a measure of elasticity). Additionally, the oleogel’s gumminess, which means its elements’ ability to stick together, also weakened as β-carotene levels increased. This effect likely occurs because β-carotene interacts with the oleogelators (beeswax) and the oil phase (canola oil), limiting the esters’ ability to create binding sites essential for maintaining the gel structure [51]. Martins et al. [88] investigated the physical configuration of beeswax oleogels incorporated with β-carotene and demonstrated how its inclusion impacts the gel structure and stability. The study utilized polarized light microscopy to examine the microstructure, revealing that the addition of β-carotene led to a more uniform crystalline network compared to oleogels without β-carotene. DSC was employed to analyze thermal transitions. β-Carotene-enriched oleogels had increased melting and crystallization temperatures, which reflects a stronger crystalline network. Additionally, small-angle X-ray scattering indicated that β-carotene incorporation reduced crystal packing heterogeneity and strengthened the gel structure. Rheological analysis revealed enhanced viscoelastic properties, with higher storage modulus (G′) values in β-carotene-enriched oleogels. Finally, the oil-binding capacity (OBC) test demonstrated that β-carotene-enriched oleogels had greater oil retention due to the denser crystalline network. These advanced characterization techniques collectively confirmed the role of β-carotene in reinforcing the structural stability and functionality of beeswax oleogels [88].

Dent et al. [53] observed the impact of curcumin on the physical properties and crystallization network of rice bran wax (RBW) oleogels. The study utilized a TA-XT2 texture analyzer to measure gel hardness. The result showed that the inclusion of curcumin significantly increased the hardness of 10% RBW oleogels (*p* < 0.05), indicating a stronger gel structure. PLM showed that oleogels with curcumin exhibited a denser and more extensive crystal network, especially at higher RBW concentrations (6% and 10%). DSC analysis demonstrated that curcumin-enriched oleogels had higher melting enthalpy, reflecting stronger physical and chemical interactions between curcumin and RBW. Additionally, X-ray diffraction (XRD) confirmed the co-crystallization of curcumin with RBW. The increased melting enthalpy of curcumin-enriched oleogels further supports the hypothesis of stronger interactions between curcumin and the RBW matrix [53].

Encapsulation of omega fatty acids (n-3 and n-6) within oleogels can enhance their oxidative stability as they are sensitive to oxidation. They interact differently with each oleogelator, and choosing the right oleogelator can help reduce lipid oxidation, enhancing the shelf life of products like spreads and dairy substitutes. A study by Dominguez et al. [89] reported that monoglyceride (MG) (oleogelator) crystal networks enhance oil stabilization in oleogels made from various oils, including those high in n-3 and n-6 fatty acids. For example, flaxseed oil rich in n-3 fatty acids produced a network of small crystals, which improved oil retention and minimized leakage. Interestingly, flaxseed oleogels retained high OBC (oil binding capacity) even with reduced MG content, suggesting effective stabilization due to the specific crystal structure and interactions with MG crystals. When oleogels are made with n-3- or 6-rich oils, they tend to have a firmer texture [89,90]. The high unsaturation levels in n-3 or 6 fatty acids enhance the aggregation of the monoglyceride (MG) head groups, hence creating a more structured and strong gel network [89].

Fat-soluble vitamins, e.g., A, D, E, and K, integrate well with oleogels, such as ethylcellulose or wax-based oleogels. These vitamins benefit from the protective environment within the oleogel, which shields them from light, heat, and oxygen, retaining their nutritional quality and bioactivity in food products over extended storage periods. The presence of vitamin E in oleogels templated with xanthan gum and lecithin (oleogelators) was studied by Hong et al. [85]. The study utilized Fourier transform infrared (FTIR) spectroscopy to confirm the incorporation of vitamin E, which exhibited characteristic peaks at 1080 cm^−1^ due to the phenyl group of vitamin E. The OBC was assessed using centrifugation, and OBC values exceeded 99.97% for E-OGs prepared with 0.3% XG. Rheological analysis demonstrated solid-like behavior with higher storage modulus (G′) compared to loss modulus (G″), showcasing improved mechanical properties. These findings suggest that vitamin E contributes to the mechanical stability of oleogels, highlighting their potential application in functional food formulations [85].

Incorporating bioactive compounds into oleogels contributes to their nutritional profile of these oleogels and influences their physical and mechanical characteristics [86]. Bioactive compounds act as gel network modifiers depending on their specific interactions with specific oleogelators [86,87]. Consequently, they modify characteristics of bioactive-enriched oleogels, e.g., hardness, elasticity, and oil-binding capacity (OBC). For instance, β-carotene and polyphenols enhance gel hardness by strengthening the crystal network [86,88]. On the other hand, n-3 or n-6 fatty acids require protective encapsulation due to their sensitivity to oxidation [89].

## 6. Processing and Storage Stability of Bioactive Oleogels

The heating, mixing, and cooling rates of bioactive-enriched oleogels have a significant impact on their stability (e.g., structure and retention of bioactive compounds) throughout food processing. For example, high processing temperatures might break down delicate bioactive substances like carotenoids, polyphenols, and omega fatty acids, affecting the oleogel’s functional and nutritional properties [16]. High temperatures, however, may also hasten phase separation since they melt the oleogelator structure, which would reduce the stability of the oleogel matrix. Oleogels can maintain the effectiveness of bioactive compounds and their firmness by meticulously controlling temperature, particularly for bioactive-rich plant-based oils. That is why controlling temperatures is essential to enhance the functional advantages of bioactive-enriched oleogels and extend their shelf life [36].

The cooling rate in oleogel formation significantly influenced the 3D structure of oleogelators and, further, the physical properties of finished oleogels. A slower cooling rate helps to form larger, more organized crystalline structures within the gel matrix, which enhances oleogel strength and stability [91]. These larger crystals can also interlock more effectively and generate a more robust oleogel matrix with better oil and bioactive compound retention. This organized crystal network minimizes spaces between the crystalline structures and, consequently, entraps oil more efficiently, hence preventing oil migration. Moreover, a slower cooling rate exhibits higher oil binding capacity and improved oxidative stability of oleogel, which are crucial for the effective delivery and preservation of bioactive compounds [17]. Conversely, a faster cooling rate resulted in smaller, less organized crystals. These smaller crystals do not interlock effectively and may prevent the oleogelators from forming a uniform network, which, as a result, creates a weaker and more porous gel network [91]. The higher porosity of crystal structure allows greater exposure of bioactive compounds within the oleogels to the oxidative factors and reduces their oxidative stability. As a result, the oleogel shows a lower oil binding capacity, which means it has reduced structural stability and is more prone to oil leakage. For instance, Co and Marangoni, (2013) investigated the impact of cooling rate on the microstructure of 12-hydroxystearic acid (12-HSA) and its influences on the stability and mechanical properties of oleogels [92]. At a high cooling rate (30 °C/min), 12-HSA produced a small spherulitic microstructure with high branching. This formation is associated with a less organized structure, consequently showing lower storage modulus (G′) and yield stress. On the other hand, at a low cooling rate (1 °C/min), 12-HSA yielded a fibrillar structure with more organized and thicker crystal formations. The slower cooling allowed more time for crystal alignment and network strengthening [92].

The intensity of mixing in oleogel processing impacts the internal structure of oleogels by affecting the size, shape, and distribution of the oleogelator’s crystal network. For example, excessive mixing weakens the oleogel’s strength and structural stability by disrupting the delicate network. On the other hand, moderate mixing promotes a stable network, which ensures even dispersion of bioactive substances and prevents their separation at their storage [16]. That is why adequate mixing is necessary to ensure uniform distribution of oleogelators and bioactive compounds [36]. A study by Da Silva et al. [93] evaluated the impact of high-intensity ultrasound (HIU), which can simulate effects like varying agitation or mixing intensity on the physical properties of oleogels. Higher agitation (or ultrasound amplitude and duration) reduced the crystal size and formed more homogeneity, which improved the oleogel’s firmness and strength. Moreover, greater mixing intensity helped retain oil within the gel matrix by reducing larger gaps in the crystalline structure. Consequently, samples treated with higher ultrasound intensity showed reduced oil loss over time [93]. Overall, the study showed that it is possible to manipulate the physical characteristics of oleogels, such as texture, oil retention, and stability, by adjusting mixing intensity (or HIU settings). Therefore, achieving optimal processing conditions, including controlled mixing and cooling, is essential to maintaining the stability and integrity of bioactive-enriched oleogels [93].

Storage conditions, e.g., temperature, humidity, and light exposure, significantly impact the physicochemical stability and bioactive compound retention in oleogels. Elevated temperatures may promote oxidation, especially in PUFA-rich oleogels, compromising the bioactive compounds and overall quality of the oleogel. Increased temperature destabilizes the oleogel network by decreasing the firmness and higher rates of compound oxidation [94]. Lower temperatures (e.g., 5 °C) maintain better structural stability, hence preserving the physical properties and bioactive components for a longer period. At lower temperatures, the oleogel matrix retains more of its hardness and prevents phase separation [94].

A previous study by Alongi et al. [95] reported that as the storage temperature increased (at 40 °C), there was a faster degradation of bioactive compounds (hydroxytyrosol, tyrosol, and α-tocopherol) in olive-oil-based oleogels. The rate of bioactive degradation followed a consistent rate over time (zero-order kinetics), which was more evident at 40 °C. Moreover, monoglycerides and rice bran wax as oleogelators retained the firmness and structure of oleogels across storage temperatures and durations. On the other hand, the EC-based oleogel maintained its structure at 40 °C but experienced breakdown (loss of firmness and oil absorption capacity) at 20 °C and 30 °C. The study suggests that higher temperatures might stabilize the network of EC-based oleogel, while lower temperatures hinder network formation [95]. The firmness of the oleogel structure is also impacted by higher temperatures, which tend to reduce the oleogel’s structural stability and result in increased oxidation. For example, significant losses in β-carotene were observed in oleogels stored at elevated temperatures (up to 40 °C) due to the increased oxidation rate [17].

Humidity can affect the moisture content and, consequently, the textural properties, making the oleogel more prone to microbial degradation. Tanislav et al. [96] showed the effects of humidity on the structural and physicochemical stability of oleogels, with an emphasis on how moisture influences bioactive retention and overall matrix integrity. Though oleogels are hydrophobic, hydrophilic oleogelators may absorb moisture in humid environments. High humidity levels can weaken the structural stability of oleogels, which can result in partial oil release or phase separation. However, the oleogel network is critical for trapping oils and bioactive compounds within the gel structure. Bioactive compounds, especially those sensitive to moisture, such as β-carotene and polyphenols, showed reduced stability under humid storage conditions [96].

Light-sensitive bioactive substances, such as carotenoids and several vitamins, oxidize more quickly when exposed to light, emphasizing the necessity of light-protective packaging in commercial settings. The article by Martins et al. [88] investigated the impact of light and air on the physicochemical properties (oxidative stability and color changes) and bioactive retention in oleogels. Exposure to light increased the oxidative degradation of bioactive compounds in oleogels (higher malondialdehyde levels), especially when the concentration of structuring agents was low. This oxidative degradation is crucial because it impacts both the retention of bioactive compounds, such as β-carotene, and the overall quality of the oleogel. However, higher oleogelator concentrations showed better protection against light-induced oxidation. Furthermore, the color study revealed that oleogels exposed to light had a notable color shift, especially for samples containing lower quantities of oleogelators (beeswax) [88]. However, packaging oleogels in opaque, sealed containers helps further protect their nutritional and functional stability by limiting exposure to light and air. Depending on the type of bioactive and oleogelator used, storage conditions can be changed to maintain the oleogel’s texture, stability, and bioactivity over time. It is crucial to store in the dark to minimize oxidation since light exposure accelerates lipid oxidation and the degradation of bioactive substances [94].

## 7. Future Directions and Challenges

The commercial production of bioactive-enriched oleogels is a necessary next step for their incorporation into mainstream food systems. Although research on bioactive compounds and oleogels has shown promising outcomes, scaling production from the laboratory to industrial levels remains a significant challenge. The primary obstacle in scalability lies in ensuring consistent quality, texture, and bioactivity during mass production. Variability in raw materials, such as plant-based oils, oleogelators, and natural bioactive compounds, can lead to inconsistencies in the final product [97]. Moreover, temperature fluctuations, irregular processing (e.g., cooling rate and mixing intensity), and storage conditions negatively impact the oleogel network. Consequently, oleogels experience weak textural properties, reduced oil retention, and phase separation [27]. The development of standardized protocols and more efficient production technologies are required to overcome these challenges, which will also maintain the oleogel’s consistency, desired texture, and stability in food applications. However, advanced manufacturing processes (high shear mixing, 3D food printing, and continuous processing systems) could help achieve reproducibility at scale. Additionally, developing modular, automated processing lines for oleogel production may address temperature fluctuations, cooling rates, and mixing intensity, minimizing textural variations, phase separation, and oil leakage. The establishment of standardized production protocols will be vital in achieving scalability and ensuring the widespread adoption of oleogels in the food industry.

The rising consumer demand for healthier and functional foods has made considerable interest in the addition of bioactive oleogels into food products. That is why consumer acceptance is another key factor for the successful commercialization of bioactive oleogels. The consumption of bioactive compounds provides health benefits, including some disease prevention. Their encapsulation or delivery utilizing oleogels in functional foods is desired [15]. However, consumer perceptions of texture and taste remain barriers to their incorporation into food products. In designing oleogels, it is important to consider that the structuring agents strongly influence the oleogel’s structure and digestibility and bioaccessibility of bioactive compounds [73,85,88]. Addressing sensory attributes, such as reducing waxy mouthfeel in wax-based oleogels, can improve consumer acceptance. Strategies like partial replacement of saturated fats with oleogels in traditional food products have shown improvement in palatability. Moreover, public education campaigns about the health benefits (e.g., cardiovascular benefits or enhanced cognitive function) of bioactive-enriched oleogels can help in raising awareness. Consumer-focused studies assessing sensory preferences, and perceptions of health claims will provide valuable insights to guide product development. Additionally, clinical studies are another potential area for future research. These studies will assess the health impacts of bioactive-enriched oleogels and provide strong evidence to support their efficacy in improving health outcomes after their incorporation into food systems. However, clinical data on bioactive-enriched oleogels remains limited at present, and future findings will be influential in validating their nutritional and functional benefits.

Expanding the range of bioactive compounds used in oleogels represents an exciting frontier. Research should explore novel compounds with unique health benefits, such as phytochemicals for cardiovascular or cognitive health or underutilized bioactive substances from marine or plant-based sources. These advancements could facilitate specific health issues by broadening the functional applications of bioactive oleogels. The encapsulation and stability of bioactive compounds within the oleogel matrix will remain critical areas of investigation to ensure their efficacy and bioavailability over extended storage periods. Furthermore, studying interactions between oleogelators and bioactive compounds at the molecular level using advanced techniques could yield novel insights.

The utilization of bioactive oleogels in daily consumed foods presents opportunities to enhance their nutritional profile. However, an individual oleogel formulation cannot provide the required quality for a wide variety of food items because of its mechanical characteristics and mouthfeel limitations [12,26]. The mechanical characteristics and mouthfeel of oleogels depend on the oleogelator and type of oil [13,42,44]. That is why future research should focus on oleogel formulations for specific food categories by optimizing texture, taste, and shelf life. Additionally, it is essential to investigate the interaction of oleogels with different heat levels, moisture content, and mechanical pressures in food processing methods to ensure their compatibility with food manufacturing techniques. Collaborations between academia and industry could accelerate the development of oleogel formulations for specific culinary applications, ensuring functionality and consumer satisfaction.

Regulatory considerations also play a pivotal role in the commercialization of bioactive oleogels. Although there is growing interest in natural oleogelators, establishing regulatory guidelines for their use in oleogels or fat-replacement foods remains complex. The use of oleogels in food applications faces regulatory scrutiny in terms of the safety and acceptability of oleogelators [12]. Consequently, particular regulatory constraints on specific oleogelators may limit their applicability in food formulations. The satisfaction with food safety standards and regulatory approvals of oleogels in different food products are key steps for their market entry. Considering the food regulations, it is necessary for oleogels to align with food safety standards from relevant authorities to facilitate their widespread use in diverse food products. New bioactive compounds and oleogel ingredients require GRAS status. That is why addressing regulatory challenges is essential for the commercial application of bioactive oleogels.

Moreover, the production of oleogels with novel or specialized oleogelators often incurs higher costs compared to traditional fat-based products. The expenses associated with sourcing novel oleogelators and their specialized processing equipment increase the production costs of oleogels [98]. Therefore, achieving scalability while maintaining cost-effectiveness is essential for the practical and sustainable implementation of oleogels within the food industry. In the future, addressing these areas can facilitate the large-scale commercialization of bioactive oleogels and offer a wide range of applications with potential health benefits. Each challenge and research direction presents an opportunity for innovation, paving the way for oleogels to become a prominent ingredient in the functional food landscape.

## 8. Conclusions

The application of oleogels is a sustainable and nutritionally beneficial approach to lipid-based food products. Their unique three-dimensional matrix encapsulates, stabilizes, and helps in the controlled release of bioactive compounds. The incorporated bioactive compounds also have an effect on the overall physicochemical properties and storage of oleogels. Bioactive oleogels facilitate the creation of functional foods that correspond with contemporary health and wellness trends. The integration of bioactive oleogels into food items diminishes dependence on trans fats and saturated fats while enhancing their nutritional profile and sensory characteristics. They have been suitable for a wide range of food products due to their potential to mimic the texture and sensory characteristics of conventional fats. Additional research is required to enhance the formulation of bioactive-enriched oleogels. This entails the selection of optimal food-grade oleogelators, the selection of bioactive compound incorporation techniques, and the evaluation of their behavior in a variety of food matrices. Additionally, the scalability, cost-effectiveness, and regulatory compliance of bioactive oleogels will be essential for their application in commercial food products. Future work should also focus on consumer acceptance and ensuring that oleogel-based products are well-received in the marketplace.

## Figures and Tables

**Table 1:** Overview of recent developments in bioactive oleogels and their application in food products.

Oleogelators	Oil Phase	Bioactive Compounds	Incorporation Techniques of Bioactive Compound	Applied Food Products	Major Outcomes	References
Beeswax and soybean lecithin (2, 4, 6, 8, and 10%)	Soybean oil	Tea polyphenols (30 mg/mL, 0.053%)	Direct encapsulation	------	1. Reduced peroxide value (PV) and malondialdehyde (MDA) levels 2. Minor decrease in the mechanical strength 3. Extended the shelf-life of the oleogels	[37]
Beeswax (5%)	Canola oil	β-Carotene (0.025, 0.05, 0.1, 0.2, and 0.4%)	Direct dissolution	------	1. Improved the oxidative stability of the oleogel 2. Significantly lower peroxide values at 0.4% curcumin 3. Smoother and denser oleogel structure 4. Softer and less elastic oleogel	[51]
Glyceryl monostearate and tea polyphenol mixture (8%)	Soybean oil	Tea polyphenol (0.054%), and curcumin (0.1%)	Direct encapsulation and dissolution	------	1. Improved oxidative stability 2. Slightly disrupted the crystallization behavior 3. Reduced stiffness and gel strength	[52]
Rice bran wax (2, 6, or 10%)	Corn oil	Curcumin (0.1%)	Direct dissolution	------	1. Increased the density and crystallinity of the oleogel 2. Increased the hardness of oleogel 3. Higher melting enthalpy of oleogels	[53]
Sodium caseinate (2%, 3%, 4%, 5%, and 6%)	Sunflower oil	Quercetin (0.012804% max)	Emulsion-templated approach	Pound cake	1. Significantly higher antioxidant capacity 2. Improved oil binding capacity 3. Thermal stability during drying	[55]
Candelilla wax and glycerol monostearate (at 1:3 ratio, 10%)	Sunflower oil	β-Carotene (0.02%)	Direct dissolution	Muffin	1. Strong oleogel structure 2. Greater mechanical strength 3. Lower peroxide values	[56]
Carnauba wax, sitosterol/lecithin or ethyl cellulose (16%)	Peanut and soybean oil	Resveratrol (0.5%)	Gelator-assisted stabilization	------	1. Improved hardness and viscoelastic properties 2. Improved oil binding capacity 3. Enhanced thermal stability	[57]
Polyphenol-enriched egg white protein (10%)	Corn oil	Polyphenols (0.2 to 1%)	Polymer complexes	------	1. Reduced peroxide values (PV) and malondialdehyde (MDA) 2. More stable and cohesive gel structure 3. Greater hardness and resistance to deformation	[67]
Carnauba wax (5%)	Canola oil	Thyme essential oil (0.5, 1, and 2% *v*/*v*)	Direct dissolution	Chicken nuggets	1. Reduced peroxide values 2. Reduced fat uptake during deep frying 3. Retained more moisture in nuggets	[72]
Ethyl cellulose (12%) and sorbitan monopalmitate (0, 2.4, 3, 4, or 6%)	Corn oil	Curcumin (0.4%)	Direct dissolution	------	1. Reduced lipid peroxidation 2. Maintained higher oxidative stability 3. Inhibited curcumin crystallization during storage	[73]
Candelilla wax (3%)	High-oleic-acid sunflower oil	Quercetin (0.1%)	Direct dissolution	------	1. Enhanced thermal and environmental stability 2. Enhanced antioxidant properties 3. Improved structural stability	[74]
Beeswax (5%)	Sunflower oil	Polyphenols (0.034%)	Solvent-assisted dispersion	------	1. Inhibited lipid peroxidation 2. Reduced lipid hydroperoxide formation 3. Improved lipid protection	[75]
Monoglycerides (10%)	Palm oil	β-Carotene (1%)	Gelator-assisted stabilization	------	1. Improved oil retention 2. Reduced degradation during thermal processing	[76]
Candelilla wax (3%)	High-oleic-acid sunflower oil	Quercetin (0.1% in oil phase)	Bigel system	------	1. Enhanced antioxidant potential 2. Enhanced the lipid phase’s bioactivity 3. Simultaneous release of hydrophilic and lipophilic compounds	[70]
β-Sitosterol and lecithin (at ratio 4:1, 16%)	Soybean or peanut oil	Resveratrol (0.5%)	Direct dissolution	------	1. Enhanced oxidative stability 2. Produced denser and more stable internal network 3. Improved storage stability	[77]
Beeswax (7.92, 8.24, and 9.12%)	Sunflower oil, avocado oil, and linseed oil	Curcumin (0.2%)	Direct dissolution	------	1. Delayed peroxide formation 2. Reduced generation of secondary oxidation products 3. Reduced the extent of lipolysis (~10%)	[78]
Starch (4.26%) and beeswax (2.13%)	Sunflower oil	Curcumin (0.1 and 0.5%)	Emulsion-templated approach	------	1. Reduction in the peroxide index 2. Reduced the hardness but improved the structural stability during storage 3. Improved long-term stability of the oleogels	[79]
Rice bran wax (4, 6, 8, and 10%)	Soybean oil	Resveratrol (0.2%)	Emulsion-templated approach	------	1. Enhanced the oxidative stability of the oleogels 2. Enhanced the viscosity and elastic behavior of the oleogels 3. Improved thermal stability	[80]
Gelatin (10%, *w*/*v*) and gellan gum (1, 1.5, and 1.75%, *w*/*v*)	Medium-chain triglycerides	Resveratrol (1.5%)	Emulsion-templated approach	------	1. Enhanced the oxidative stability of the oleogels 2. Exhibited higher bioaccessibility of resveratrol 3. Higher storage modulus values and reduced oil leakage	[81]
Gelatin (1.2%), tannic acid (0.3%), and flaxseed gum (0.6%)	Soybean oil	Polyphenols (0.075%)	Polymer complexes	------	1. Improved network stability 2. Enhanced rheological properties 3. Increased the melting point of the oleogels	[82]
Beeswax or ethyl cellulose (11%)	A mixture of olive, linseed, and fish oils (44.39%, 37.87%, and 17.74%, respectively)	Curcumin (0.2%)	Direct dissolution	Pork burgers	1. Reduced cooking loss in some formulations 2. Improved the oxidative stability of the oleogels 3. Reduced the formation of malonaldehyde	[83]
Xanthan gum (0.32%) and soy lecithin (1.2%)	Soybean oil	Turmeric extract (curcumin) (1%)	Emulsion-templated approach	Pound cake	1. Enhanced the oxidative stability of oleogels 2. Exhibited a controlled release of curcumin 3. Reduced the hardness of pound cakes	[84]
Xanthan gum (0.3%) and soy lecithin (2.0%)	Soybean oil	Vitamin E (n.m.)	Emulsion-templated approach	Pound cake	1. Enhanced the oxidative stability of oleogels 2. Higher bioaccessibility of vitamin E 3. Increased porosity of pound cakes	[85]

The concentration (%) of oleogelators, liquid oil, and bioactive compounds is *w*/*w* if they are not mentioned in another way. n.m. = not mentioned.

## Data Availability

Not applicable.
